# A Compendium of Co-regulated Protein Complexes in Breast Cancer Reveals Collateral Loss Events

**DOI:** 10.1016/j.cels.2017.09.011

**Published:** 2017-10-25

**Authors:** Colm J. Ryan, Susan Kennedy, Ilirjana Bajrami, David Matallanas, Christopher J. Lord

**Affiliations:** 1 School of Computer Science, University College Dublin, Dublin 4, Ireland; 2 Systems Biology Ireland, School of Medicine, University College Dublin, Dublin 4, Ireland; 3 The Breast Cancer Now Toby Robins Breast Cancer Research Centre and CRUK Gene Function Laboratory, The Institute of Cancer Research, London SW3 6JB, UK

**Keywords:** cancer, breast cancer, proteomics, protein complexes, post-transcriptional control, driver gene mutations, proteogenomics, E-cadherin

## Abstract

Protein complexes are responsible for the bulk of activities within the cell, but how their behavior and abundance varies across tumors remains poorly understood. By combining proteomic profiles of breast tumors with a large-scale protein-protein interaction network, we have identified a set of 285 high-confidence protein complexes whose subunits have highly correlated protein abundance across tumor samples. We used this set to identify complexes that are reproducibly under- or overexpressed in specific breast cancer subtypes. We found that mutation or deletion of one subunit of a co-regulated complex was often associated with a collateral reduction in protein expression of additional complex members. This collateral loss phenomenon was typically evident from proteomic, but not transcriptomic, profiles, suggesting post-transcriptional control. Mutation of the tumor suppressor E-cadherin (*CDH1*) was associated with a collateral loss of members of the adherens junction complex, an effect we validated using an engineered model of E-cadherin loss.

## Highlights

• Identified a set of 285 complexes coherently expressed across breast tumor proteomes
• Identified complexes over- or underexpressed in specific breast cancer subtypes
• Loss of one subunit often associated with reduced expression of a co-regulated complex
• E-cadherin (*CDH1*) mutation causes reduced expression of an adherens junction complex

## Graphical Abstract

**Figure:**
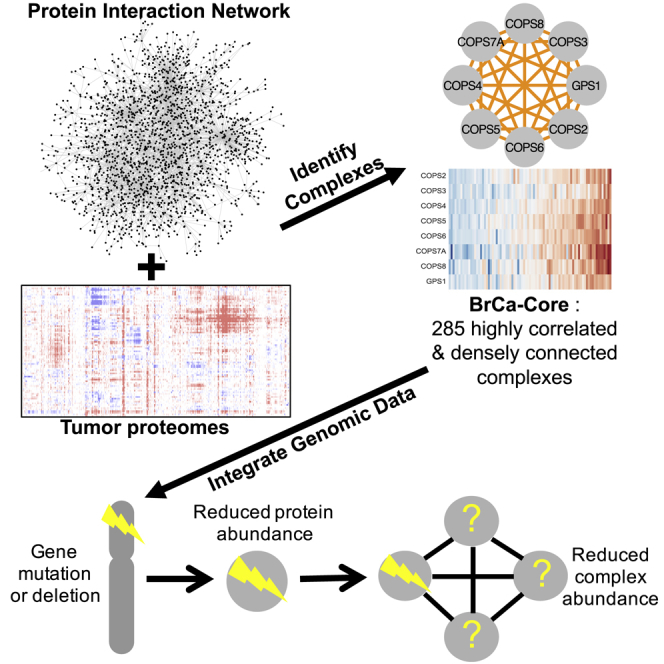

## Teaser

Ryan et al. develop an approach to identify co-regulated protein complexes from breast tumor proteomic profiles and demonstrate that genomic loss of one subunit is often associated with a reduction in the protein expression of an entire complex.

## Introduction

Multi-subunit protein complexes are responsible for the bulk of the functionality of the cell (Alberts, 1998, Hartwell et al., 1999). Despite their importance to cellular function, relatively little is known about how the functionality and expression of protein complexes are altered in different cancer subtypes or in individual cancer patients. Recent examples in breast cancer suggest that even “housekeeping” complexes traditionally thought of as constitutively active and essential in all cell types, such as the ribosome and the spliceosome, may become differentially expressed or differentially essential in specific contexts (Hsu et al., 2015, Pozniak et al., 2016). Consequently, there is a great need to characterize the altered behavior of protein complexes in cancer.

Largely for technical and economic reasons, the large-scale molecular profiling of tumors performed over the past decade has focused on characterizing changes at the genomic and transcriptomic level. Transcriptomic measurements are often used as a proxy measurement for protein expression, but most genes display only a moderate correlation between their mRNA and protein expression levels (Liu et al., 2016, Vogel and Marcotte, 2012). Moreover, this correlation varies considerably between genes, with members of large protein complexes such as the ribosome and spliceosome reported to have significantly lower mRNA-protein correlation than average (Mertins et al., 2016, Zhang et al., 2016). Taken together, these observations suggest that efforts to understand altered protein complex functionality must rely on more direct measurements of protein expression. Recently, advances in mass spectrometry have enabled the quantification of thousands of proteins across large numbers of samples (Mertins et al., 2016, Pozniak et al., 2016, Tyanova et al., 2016). These datasets permit, for the first time, large-scale assessment of the behavior of protein complexes across different tumor samples and between different tumor types. Here, we develop an approach to identify co-regulated protein complexes from tumor proteomic profiles and characterize the expression of these protein complexes across 77 breast tumor proteomes (Mertins et al., 2016).

## Results

### Similarity of Co-expression Profiles Is Highly Predictive of Protein Complex Membership

We first wished to assess whether known protein complexes are coherently regulated across tumor proteomes. Using the CORUM manually curated set of human protein complexes (Ruepp et al., 2010) and 77 protein expression profiles from the Cancer Genome Atlas (TCGA) breast cancer proteomics project (Mertins et al., 2016), we assessed the relationship between the similarity of protein expression profiles and the likelihood of two proteins belonging to the same protein complex (Figure S1A). In comparison with the correlation observed using mRNA expression profiles, protein expression profiles were more predictive of co-complex membership (Figure S1A). This observation is consistent with recent work that found, using tumor profiles, that protein co-expression was more predictive of general functional similarity than mRNA co-expression (Wang et al., 2017). We assessed whether reducing molecular heterogeneity, by calculating protein co-expression on samples from a single breast cancer subtype, would improve our ability to predict protein complex membership from protein expression and found no obvious improvement (Figures S1B and S1C).

Although co-expression calculated over the same number of samples suggested a significant advantage of proteomic profiles over mRNA profiles, the number of existing tumors with mRNA profiles far exceeds the number with proteomic profiles. We found that even with all TCGA breast tumor samples included (∼14 times as many mRNA profiles as proteomic profiles), the proteomic profiles still outperformed mRNA in predicting co-complex membership (Figure S1A). This suggested that post-transcriptional processes such as translation and protein turnover may significantly contribute to maintaining the stoichiometry of protein complexes. Consistent with this, we found that the median Pearson's correlation between mRNA and protein expression for genes annotated in CORUM complexes is significantly lower than that for all other genes (0.36 for genes in complexes versus 0.4 for all other genes; Mann-Whitney, p < 1.5 × 10^−6^) suggesting increased post-transcriptional control of protein complex subunits.

**Figure 1:**
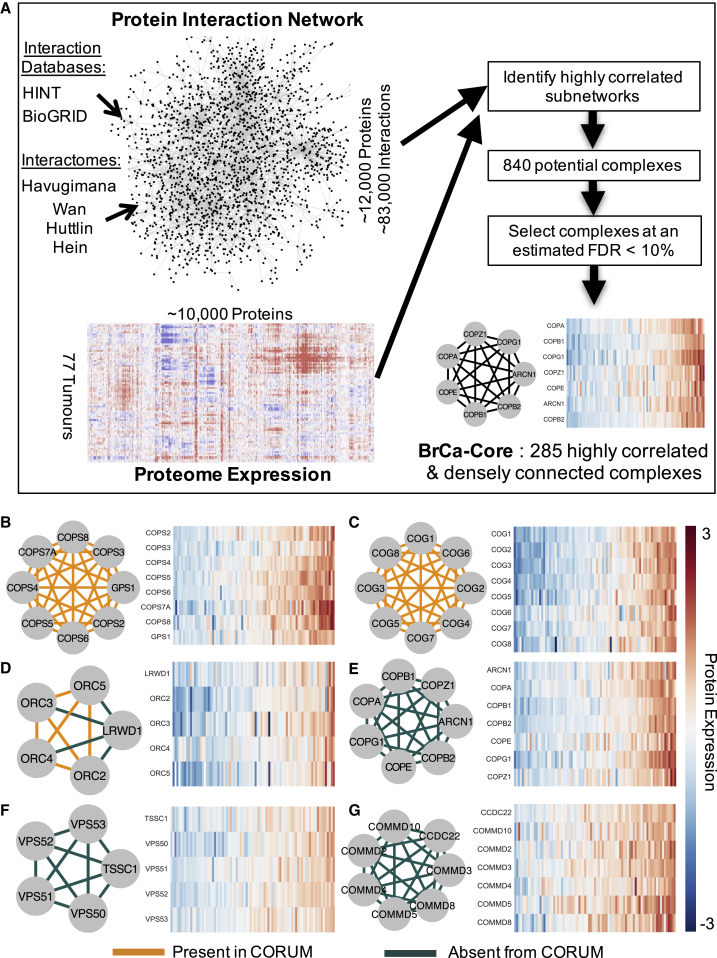
BrCa-Core Complex Discovery (A) An integrated protein-protein interaction network is combined with tumor proteomic profiles to identify sets of densely connected proteins that display correlated expression profiles across tumor proteomes. By comparing the results with those derived from randomly relabeled protein interaction networks, we can estimate the false discovery rate (FDR). The BrCa-Core set contains 285 complexes at an estimated FDR of 10%. (B) BrCa-Core 17: the COP9 signalosome. The heatmap on the right shows protein expression of all subunits across 77 breast tumor proteomes. These have been sorted based on the mean abundance of all subunits. (C) BrCa-Core 14: the conserved oligomeric Golgi (COG) complex. (D) BrCa-Core 47 contains the ORC2–5 complex found in CORUM with the addition of LRWD1. (E) BrCa-Core 25, the COPI complex. (F) BrCa-Core 48, the EARP complex with the recently identified EARP interactor TSSC1. (G) BrCa-Core 26, the Commander complex.

While in general the expression of different subunits within the same CORUM complex was highly correlated, this was not the case for all complexes examined, suggesting that not all complexes are coherently regulated to a similar degree in breast cancer (Figure S1D). Moreover, visual exploration of the expression data suggested that there were highly correlated groups of proteins corresponding to known complexes that were absent from the CORUM curated set. With these issues in mind, for further analysis we elected to use a data-driven approach to identify protein complexes coherently regulated in breast cancer (Figure 1A).

### A Compendium of Protein Complexes Co-regulated in Breast Tumors

We hypothesized that by integrating large-scale protein-protein interaction networks with proteomic profiling, we could identify protein complexes coherently regulated in breast tumors. We first constructed a large network of protein-protein interactions by integrating literature-curated interaction databases (Chatr-Aryamontri et al., 2016, Das and Yu, 2012) with recently generated large-scale high-throughput protein interaction maps (Havugimana et al., 2012, Hein et al., 2015, Huttlin et al., 2015, Wan et al., 2015) (Figure 1A). As expected, we found that integrating this protein interaction network with co-expression improved our ability to predict co-complex membership (Figure S1E, STAR Methods). To identify sets of genes that are densely connected on this network and display highly correlated expression profiles across multiple tumor samples, we developed a constrained clustering approach that integrated the protein-protein interaction network with proteomic expression profiles from 77 breast tumors (Mertins et al., 2016) (Figure 1A, STAR Methods). Using this approach, we identified a high-confidence set of 285 complexes encompassing 1,116 distinct proteins (Figure S1 and Table S1). We refer to this set of complexes throughout as BrCa-Core 1–285. The identified complexes range in size from 2 subunits to 43 subunits (mean size, 3.9) with the largest complex corresponding to the cytosolic ribosome (BrCa-Core 1). Just under half of the BrCa-Core complexes (n = 138) significantly overlap with literature-curated complexes annotated in CORUM (adjusted p < 0.05), including the COP9 signalosome (Figure 1B, BrCa-Core 17) (Seeger et al., 1998) and the conserved oligomeric Golgi (COG) complex (Figure 1C, BrCa-Core 14) (Ungar et al., 2002). Some of the BrCa-Core complexes encapsulated protein complexes already annotated in CORUM along with additional subunits; for example, BrCa-Core 47 included the CORUM-annotated origin-recognition 2–5 complex (ORC 2–5) (Dhar and Dutta, 2000) with the addition of LWRD1, which interacts with the ORC complex and stabilizes binding of the complex to chromatin (Shen et al., 2010) (Figure 1D). Complexes identified in BrCa-Core but absent from the CORUM human complex set include the COPI-vesicle coat complex (Figure 1E, BrCa-Core 25), a variant of the endosome-associated recycling protein (EARP) complex that includes all four EARP subunits along with the more recently identified EARP interactor TSSC1 (Gershlick et al., 2016, Schindler et al., 2015) (Figure 1F, BrCa-Core 48), and a complex containing the majority of subunits of the newly identified “Commander” (COMMD/CCDC22) complex (Figure 1G, BrCa-Core 26) (Starokadomskyy et al., 2013) recently shown to be highly conserved across metazoans (Wan et al., 2015).

The majority of BrCa-Core complexes have significant overlap with specific Gene Ontology Cellular Component and Biological Process terms, suggesting common localization and functionality, respectively (208 complexes enriched in GO-CC terms, 235 enriched in GO-BP terms, both at adjusted p < 0.05) (Table S1). Like known protein complexes, pairs of proteins assigned to the same BrCa-Core complex were significantly more likely than random protein pairs to be frequently mentioned together in the literature (odds ratio, 175; p < 1 × 10^−16^, Fisher's exact test) and to display similar patterns of conservation across species (odds ratio, 277; p < 1 × 10^−16^, Fisher's exact test).

As our method exploited the correlation between protein expression profiles to identify complexes, we expected the average correlation across the TCGA proteomes within the BrCa-Core complexes to be high. This was indeed the case; the observed correlation (0.63) was higher than the average of pairs in our integrated protein interaction network (PPI pairs, 0.12) or pairs within CORUM complexes (0.20). To rule out the possibility that we were merely overfitting our results to a single proteomics dataset, we assessed whether the same higher correlation could be observed in two additional breast tumor proteomic datasets (Pozniak et al., 2016, Tyanova et al., 2016). In both Pozniak et al. (BrCa-Core, 0.28; CORUM, 0.14; PPI pairs, 0.10) and Tyanova et al. (BrCa-Core, 0.32; CORUM, 0.19; PPI pairs, 0.12), we found higher average correlation for BrCa-Core pairs.

The tendency of pairs of proteins within the same complex to display similar phenotypes when inhibited has been well established in the literature (Sharan et al., 2007, Wang and Marcotte, 2010). To assess whether the BrCa-Core complexes also displayed a similar tendency, we analyzed the results of a recently published large-scale short hairpin RNA (shRNA) screen in 77 breast tumor cell lines (Marcotte et al., 2016). We expected that shRNAs targeting members of the same complex would display correlated essentiality profiles (i.e., would inhibit tumor cell lines in a similar fashion), and we found that this is indeed the case (BrCa-Core, 0.24; CORUM, 0.07; PPI pairs, 0.06).

The BrCa-Core complexes contain complete or partial coverage of 538 CORUM complexes (average percent of CORUM complex members included in the corresponding BrCa-Core complex is 57%) corresponding to 39% of the CORUM complexes represented in the proteomic dataset (538/1,380). We note that this is larger than the number of BrCa-Core complexes that significantly overlap with CORUM complexes (138) due to the heavily overlapping nature of CORUM complexes. In CORUM, the average protein belongs to three distinct complexes, while by design, in BrCa-Core, each protein was assigned to a single complex based on highly correlated expression with other members. The subset of CORUM co-complex pairs we identify in BrCa-Core have higher average protein co-expression (average correlation 0.65) than those not identified in BrCa-Core (average correlation 0.16). One explanation for this is that we have preferentially identified complex cores or modules (Gavin et al., 2006). Many protein complexes exist in multiple isoforms, with the exact composition varying across cell types and conditions. Previous work in yeast has suggested that the subunits of protein complexes can be divided into two groups: cores (proteins found in the majority of complex isoforms) and attachments (proteins found in a small number of isoforms) (Gavin et al., 2006). Some pairs of attachment proteins are often found together in multiple complexes, and these have been referred to as “modules” (Gavin et al., 2006). Consistent with BrCa-Core preferentially identifying cores or modules, we found that pairs of proteins annotated together in two or more CORUM complexes were more likely to be identified together in a BrCa-Core complex (odds ratio, 1.9; p < 1 × 10^−16^, Fisher's exact test) as were pairs always found in the same CORUM complex (odds ratio, 6.6l p < 1 × 10^−16^, Fisher's exact test).

### Differential Expression of Protein Complexes in Breast Cancer Subtypes

At the molecular level, breast cancer is a very heterogeneous disease, with each tumor displaying a unique genetic and epigenetic profile. Despite this heterogeneity, molecular biomarkers can be used to classify tumors with similar molecular profiles into subtypes that display different survival outcomes and different responses to targeted therapies (Onitilo et al., 2009, Perou et al., 2000, Sorlie et al., 2001). The biomarkers used most commonly in the clinic are estrogen receptor (ER), progesterone receptor (PR), and human epidermal growth factor receptor 2 (ERBB2/HER2), often measured using immunohistochemistry (IHC) (Onitilo et al., 2009). To better understand how breast cancer subtypes might influence protein complexes (and vice versa), we assessed the relationship between BrCa-Core protein complex expression and IHC-defined subtypes. To enable the identification of reproducible associations between subtypes and protein complex abundance, we focused on those subtypes with reasonable representation in both the TCGA dataset and the dataset of Tyanova et al. (2016): HER2+ (ER^−^/PR^−^/HER2^+^), ER^+^ (ER^+^/PR^+^/HER2^-^), and triple negative (ER^−^/PR^−^/HER2^−^).

**Figure 2:**
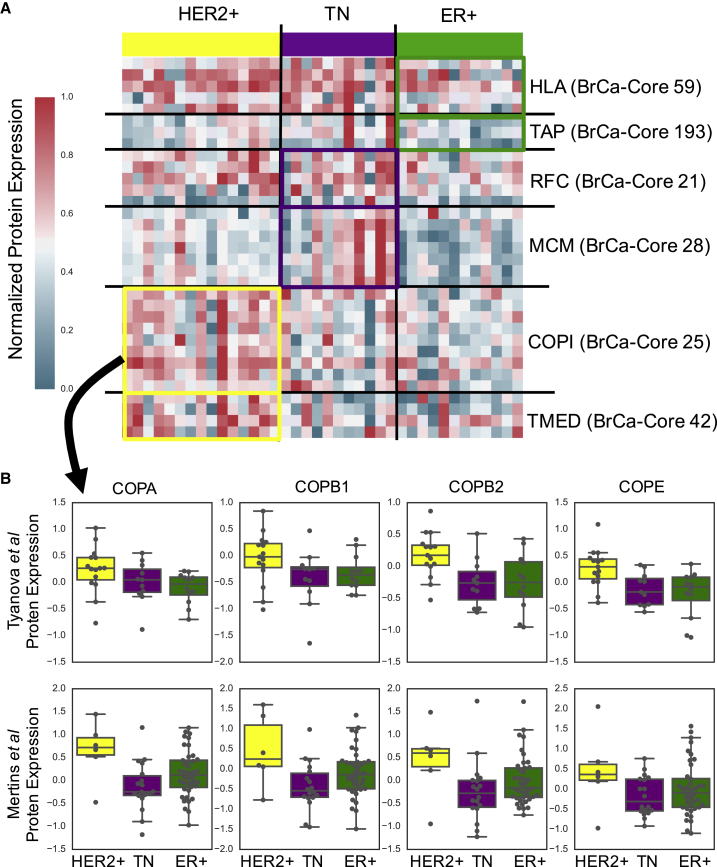
Subtype-Specific Complex Expression (A) Heatmap displaying protein expression levels of specific BrCa-Core complexes. Tumor samples are grouped according to subtype (using IHC markers), indicated on top of the heatmap. Genes are grouped into specific complexes indicated on the right of the heatmap. Shown are the expression levels taken from Tyanova et al., 2016 (used for validation). These expression levels have been normalized such that the maximum expression level is 1 and minimum is 0. Heatmap for the discovery dataset (Mertins et al., 2016) is shown in Figure S2A. Complexes differentially expressed in specific subtypes are highlighted with boxes colored to match the subtype they are differentially expressed in. (B) Boxplots displaying the subtype-specific protein expression levels of selected subunits of the COPI complex (BrCa-Core 25) in the Tyanova et al. dataset (top) and TCGA dataset (bottom). These boxplots show median and interquartile range and are colored according to sample subtype (matching Figure 2A).

Using the TCGA dataset and the BrCa-Core complexes, we discovered 80 associations between subtype and complex abundance at a false discovery rate (FDR) of 10% (Table S2, Figure 2). At the same FDR threshold, we found seven associations using the CORUM complex set, highlighting the advantage of using co-regulated BrCa-Core complexes for this analysis. Due to differences in coverage of protein complex subunits, not all of the 80 associations could be tested in the Tyanova et al. dataset. Of the 58 associations that could be tested, 27 were observed at the same FDR of 10% (Table S2). In general, the effect sizes and directions across the two datasets were highly correlated (Spearman's r = 0.68, p < 1 × 10^−8^), suggesting that with larger sample sizes, additional associations between subtype and complex abundance could be replicated. Examples of replicated differentially expressed complexes are presented in Figures 2 and S2. Triple-negative breast tumors were associated with increased expression of a number of complexes involved in DNA replication, including the replication factor C complex (BrCa-Core 21) and the MCM complex (BrCa-Core 28) (Figures 2A and S2). Different members of the MCM complex (MCM2 and MCM4) have previously been identified as markers of proliferation, associated with poorer survival outcomes in breast cancer and shown to have higher expression in ER-negative breast tumors (Joshi et al., 2015, Kwok et al., 2015). ER^+^ tumors were associated with decreased expression of two complexes involved in antigen processing (BrCa-Core 59 and 193) consistent with data suggesting that expression of antigen presentation human leukocyte antigen molecules is lower in the ER^+^ subtype (Chung et al., 2017, Lee et al., 2016). HER2^+^ tumors were associated with increased expression of two complexes involved in Golgi-transport-associated vesicle coating (BrCa-Core 25 and 42). It is not immediately obvious why *HER2* amplification would be associated with an increased expression of complexes involved in vesicle transport, but the association is evident across both patient cohorts (Figures 2B and S2, Table S2).

### The Impact of Subunit Loss on Protein Complex Expression

**Figure 3:**
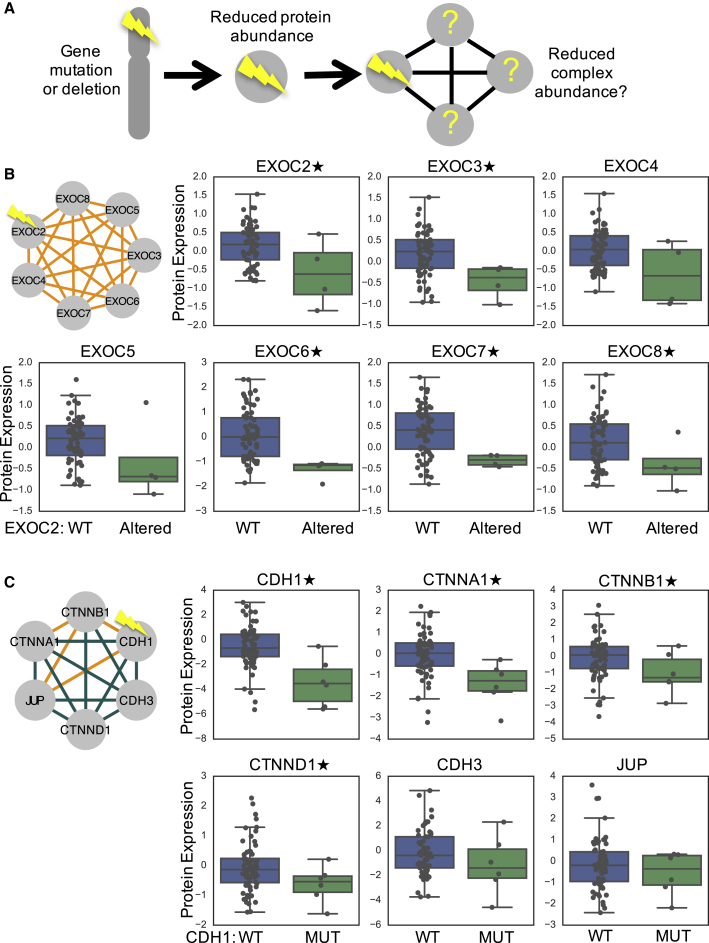
Subunit Loss Is Associated with a Reduction in Protein Complex Expression (A) Model displaying a potential series of events; mutation or deletion of one subunit is associated with reduced protein abundance of that subunit and potentially a reduction in expression of the entire complex. (B) Mutation or deletion of *EXOC2* is associated with a reduction in protein abundance of the exocyst complex (BrCa-Core 27). Boxplots display the protein abundance of different subunits partitioned according to EXOC2 status. Each boxplot shows the median and interquartile range. Genes marked with a star indicate those whose proteomic abundance is significantly lower (one-sided Mann-Whitney test, p < 0.05) in samples with *EXOC2* mutation/deletion. (C) *CDH1* mutation is associated with a reduction in protein expression of an adherens junction complex (BrCa-Core 30). Legend as for (B).

An implication of highly correlated protein expression within a protein complex is that loss of protein expression of one subunit might frequently be associated with reduced protein expression of other co-regulated complex subunits (Figure 3A). Such a reduction in expression may occur through reduced transcription, reduced translation, or an increase in protein degradation. Consequently, genetic events that reduce protein expression of one subunit, such as mutation or deletion, may be associated with a collateral reduction (in *trans*) of protein expression of other subunits or indeed the entire complex (Figure 3A). To test whether this is the case, we first focused on genes subject to homozygous deletion or mutation, reasoning that they might cause the most profound effects on protein expression. We identified five genes that are members of BrCa-Core complexes whose mutation or homozygous deletion is associated with a nominally significant (p < 0.05, Mann-Whitney U test) reduction in expression of their encoded proteins (*CDH1* (*E-cadherin*), *PBRM1*, *CYFIP2*, *GLUD1*, *EXOC2*). We then asked whether mutation or deletion of these genes was also associated with overall reduction in protein expression of the complex that they belong to. In all five cases, we found that loss of one subunit was associated with a reduction in the protein expression of additional complex subunits. For instance, homozygous deletion or mutation of *EXOC2* was associated with decreased proteomic abundance of EXOC2 and an overall reduction in the protein expression of multiple members of the exocyst complex (BrCa-Core 27) (Matern et al., 2001) to which it belongs (Figure 3B). While loss of *EXOC2* was also associated with a reduction of EXOC2 mRNA expression, no reduction was observed for other protein complex subunits at the mRNA level (Figure S3A), suggesting that the reduction in protein expression levels is caused by post-transcriptional mechanisms. Furthermore, the correlation between complex subunits was higher at the protein than mRNA level (Figure S3B), suggesting these post-transcriptional mechanisms may contribute to the coherent protein expression of the complex.

While mutations or homozygous deletions of complex subunits are relatively rare, hemizygous (single copy) deletions in tumors are frequent, and the majority of BrCa-Core member genes were hemizygously deleted in three or more tumor samples (1,053/1,116 genes). We identified 308 BrCa-Core complex members whose hemizygous deletion was associated with a reduction in the expression of their encoded protein (Mann-Whitney, p < 0.05; Table S3). The majority of these (94%, 290 genes) were also associated with a reduction in mRNA expression of their encoded genes at the same significance threshold. We then tested whether these 308 genes were associated with an overall reduction in the protein expression of their associated complex (see STAR Methods) and found that 102 genes were at an FDR of 10% (Table S3). To ensure this reduction was not merely due to co-deletion of complex members on the same chromosome, we excluded gene pairs located on the same chromosome for this analysis. Of the 102 associations, only 6 were associated with a reduction in mRNA expression of their associated complex at an FDR of 10% (Table S3). This suggests that although hemizygous deletion frequently causes a reduction in both mRNA and protein levels of the encoded protein, the impact upon other members of the complex is typically only observed at the protein level. A striking example involves the COP9 signalosome (BrCa-Core 17, Figure S4A); hemizygous loss of *COPS3* is associated with a reduction in the protein expression of all subunits (Figure S4B), but only the mRNA expression of COPS3 itself (Figure S4C). As with the exocyst complex, COP9 subunits were more highly correlated at the protein (Figure S4D) than mRNA level (Figure S4E).

### E-cadherin Loss Causes Reduced Expression of Adherens Junction Complex Members

**Figure 4:**
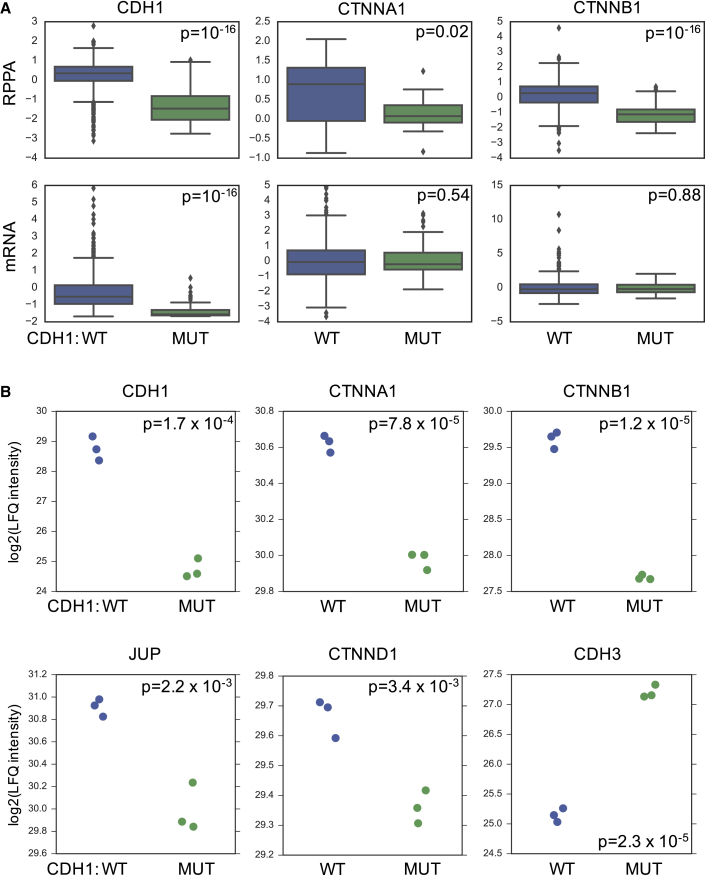
E-cadherin Loss Is Associated with Reduced Expression of an Adherens Junction Complex (A) In tumor samples, *CDH1* mutation is associated with a decrease in mRNA and protein expression of CDH1 but only of protein expression for CTNNA1 and CTNNB1. All expression and RPPA measurements are Z scores. Boxplots show median and interquartile range. p values calculated using a Mann-Whitney test. mRNA measurements for all three genes were available for 992 tumors, RPPA data for CDH1, and CTNNB1 were available for 760 tumors, while RPPA data for CTNNA1 were available for only 64 tumors. (B) Protein expression measured in a pair of isogenic MCF7 cell lines that differ by *CDH1* status. Shown are the log2 label-free quantification intensities. p values are calculated using a two-sided heteroscedastic t test.

Loss of E-cadherin is a major driver event in breast cancer, with its coding gene *CDH1* mutated in ∼11% of all breast tumors and over 50% of invasive lobular breast tumors (Berx et al., 1995, Ciriello et al., 2015, Michaut et al., 2016). Our analysis identified that mutation of *CDH1* was associated with a decreased abundance of both the E-cadherin protein and additional members of an adherens junction complex to which it was assigned in BrCa-Core (BrCa-Core 30) (Figures 3C and S3C). All proteins in this complex have highly correlated protein expression with E-cadherin (average Pearson's correlation 0.65; Figure S3D) and four of the complex subunits have a significant (Mann-Whitney, p < 0.05) decrease in expression in *CDH1* mutant samples (Figure 3C). In contrast, the average mRNA correlation of all subunits with CDH1 was low (Pearson's correlation, 0.08) with one subunit (CTNNB1) displaying weakly negative correlation with E-cadherin (Figure S3D). None of the subunits other than E-cadherin itself display a significant relationship between *CDH1* mutation status and mRNA expression (all Mann-Whitney, p > 0.05; Figure S3C). Three of the proteins in this complex (E-cadherin/CTNNA1/CTNNB1) have also been measured in a larger sample size using the RPPA method, permitting us to assess the association between *CDH1* mutation and protein abundance measured using an orthogonal approach. Using the RPPA data, we again found that *CDH1* mutation was associated with a significant reduction in abundance of all three proteins (Figure 4A) but only the mRNA of CDH1 itself (Figure 4A).

A limitation of our analysis of tumor proteomes is that it identifies correlative rather than causal associations; it demonstrates that mutation of *CDH1* is associated with reduced expression of other E-cadherin-associated subunits, but it does not demonstrate a causal effect. It is of course possible that some additional factor causes reduction in expression of the entire adherens junction complex rather than the mutation of a single subunit such as *CDH1*. To establish causality, we used mass spectrometry to measure differential protein expression in a pair of isogenic breast cancer cell lines (MCF7) with CRISPR-Cas9 engineered *CDH1* loss (STAR Methods). We recently generated a series of *CDH1* mutant clones in the MCF7 cell line (I.B. et al., unpublished data) and selected one for further study that exhibited loss of E-cadherin protein expression (Figure S5, STAR Methods). We performed label-free protein quantification of whole protein lysates in parental (MCF7 E-cadherin wild-type) and E-cadherin defective daughter cells, resulting in the quantification of ∼5,100 proteins (Table S4, STAR Methods). We found 91 proteins with significantly lower protein abundance in the E-cadherin defective model (p < 0.005; FDR, ∼8%) including five of the six adherens junction complex subunits (E-cadherin, CTNNA1, CTNNB1, CTNND1, JUP) (Figure 4B), suggesting that *CDH1* mutation plays a causative role in the reduction of their protein abundance. In contrast to what we observe in the tumor proteomes, in the MCF7 E-cadherin null model, we observed an increase in the expression of CDH3 (P-cadherin) (Figure 4B), perhaps an example of “cadherin switching” specific to this model (Cavallaro et al., 2002, Wheelock et al., 2008). The decreased abundance of five of the six BrCa-Core adherens junction complex members in the MCF7 model was a significant enrichment over random expectation (odds ratio, 280; p = 10^−8^, Fisher’s exact test). To test whether our approach missed additional collateral loss events associated with *CDH1* mutation, we assembled a list of 95 E-cadherin protein-protein interaction partners from CORUM (18 co-complexed subunits), BioGRID (89 protein-protein interaction partners), and HINT (15 co-complex interaction partners). Aside from the five members of the adherens junction complex in BrCa-Core, none of the known E-cadherin interaction partners displayed a significant reduction in protein abundance in the E-cadherin-defective model. This suggested that our data-driven approach effectively identified the specific subunits of the adherens junction complex whose expression is reduced by *CDH1* mutation in breast cancer.

## Discussion

We found that, in general, correlation between protein expression profiles predicts co-complex membership better than correlation between mRNA expression profiles. One factor that contributes to this improved correlation is the collateral loss phenomenon we observe; when one subunit of a complex is lost via deletion or mutation, a collateral loss in the protein expression of additional complex members is observed. This collateral loss is typically not observed at the mRNA level, and consequently complexes that experience collateral loss display higher correlation at the protein than mRNA level. There are likely many other factors that contribute to maintaining the coherent expression of protein complexes across tumors, including dosage compensation of copy-number-amplified genes (Geiger et al., 2010, Stingele et al., 2012).

We have not addressed here the mechanisms responsible for the collateral loss phenomenon, although the observation that the reduction in protein expression levels is not evident at the mRNA level suggests post-transcriptional mechanisms must be responsible. Perhaps the simplest explanation is that loss of one subunit prevents a complex from assembling, and consequently there is an increase in the proteasomal degradation of unbound subunits. Our analysis of hemizygous deletions suggests that complete loss of protein expression is not necessary for the collateral loss phenomenon. Similarly, we note that work in mice suggests that regulatory mutations that affect the mRNA expression of individual protein complex subunits may also cause a collateral loss in the expression of their interaction partners (Chick et al., 2016).

We note we did not always observe perfect agreement between the genotype calls in tumors and protein expression; in some instances, copy-number analysis suggested a homozygous deletion but substantial protein expression was still observed. This could reflect errors in calling the deletions, limitations of mass spectrometry protein identification, or simply heterogeneity between the portion of the tumor sample assessed for proteomic profiling and the portion assessed for genotypic profiling.

We have exclusively focused on the behavior of coherently expressed protein complexes across breast tumor samples. This approach has a number of advantages; in particular, it allows us to see how different complexes behave as a single unit within molecularly defined groups of tumors. A disadvantage of this approach is that we cannot identify when different variants/isoforms of a protein complex become more or less abundant in specific conditions. We have overlooked such events here, but recent work in cancer cell lines and mouse fibroblasts suggest that they may be relatively common and merit further investigation (Ori et al., 2016).

We expect that the BrCa-Core complexes will be useful for the analysis of additional proteomic and functional datasets and make the full list of complexes available in Table S1. We also anticipate that the complex identification approach described here will be useful for the analysis of other large-scale proteomic datasets, such as those from other tumor or cell line profiling projects, and we make our code available to facilitate such efforts.

## STAR★Methods

### Key Resources Table

**Table:** 

REAGENT or RESOURCE	SOURCE	IDENTIFIER
**Antibodies**		

E-cadherin	Cell Signalling	24E10; RRID: AB_10828126
ACTIN	Santa Cruz	I-19

**Chemicals, Peptides, and Recombinant Proteins**		

Lysl Endopeptidase	Wako	Cat#125-05061
Sequencing grade modified Trypsin	Promega	Cat#V5111
1.8μ 120Å UChrom C18 packing material	NanoLCMS Solutions	Cat#81003

**Critical Commercial Assays**		

BCA Protein Assay Kit	Pierce	Cat#23225
Edit-R CRISPR-Cas9 Synthetic tracrRNA	Dharmacon –GE Lifesciences	U-002005-05
Edit-R Predesigned crRNA	Dharmacon –GE Lifesciences	CM-HUMAN-XX-0002
Edit-R Cas9 Expression Plasmids	Dharmacon –GE Lifesciences	U-004100-120

**Deposited Data**		

MCF7 CDH1 +/- proteomic profiles	This Study	PRIDE: PXD007543

**Experimental Models: Organisms/Strains**		

MCF7 Cell Line	ATCC	CAT#HTB-22; https://www.lgcstandards-atcc.org/Products/All/HTB-22.aspx

**Software and Algorithms**		

Python version 2.7	Python Software Foundation	https://www.python.org/; RRID:SCR_008394
gProfiler	Reimand et al., 2016	http://biit.cs.ut.ee/gprofiler/
BrCa-Core Code	This study	https://github.com/cancergenetics/brca-core
MaxQuant version 1.5.0.25	Cox and Mann, 2008	http://www.maxquant.org

**Others**		

Vivacon 500 30,000 MWCO	Sartorius	CAT#VN01H22
Breast Cancer Cell Line shRNA data	Marcotte et al., 2016	https://github.com/neellab/bfg/blob/gh-pages/data/shrna/breast_zgarp.txt.zip?raw=true
HINT database	Das and Yu, 2012	http://hint.yulab.org/;RRID:SCR_002762
BioGRID database	Chatr-Aryamontri et al., 2016	https://thebiogrid.org; RRID:SCR_007393
CORUM database	Ruepp et al., 2010	http://mips.helmholtz-muenchen.de/corum/; RRID:SCR_002254
STRING interactions	Szklarczyk et al., 2017	http://string-db.org/download/protein.links.v10/9606.protein.links.v10.txt.gz; RRID:SCR_005223
cBioPortal	Gao et al., 2013	http://www.cbioportal.org/; RRID:SCR_014555
TCGA Breast Cancer Proteomics data	Mertins et al., 2016	Table S3
Tyanova et al Breast Cancer Proteomics data	Tyanova et al., 2016	Dataset S2
Pozniak et al Breast Cancer Proteomics data	Pozniak et al., 2016	Table S2

### Contact for Reagent and Resource Sharing

Further information and requests for resources and reagents should be directed to and will be fulfilled by the Lead Contact, Colm J. Ryan (colm.ryan@ucd.ie).

### Experimental Model and Subject Details

MCF7 cell line are derived from a female breast tumour and were grown in DMEM (Gibco) supplemented with 10% fetal bovine serum (Gibco) and 1% L-glutamine (Gibco).

### Method Details

#### MCF7 E-cadherin Defective Clone Selection

The *CDH1* gene in MCF7 cells was CRISPR-Cas9 mutagenised using the Edit-R-CRISPR-CAS9-gene engineering kit (GE Dharmacon) according to the supplier’s instructions. A crRNA sequence targeting exon 7 of *CDH1* was used. Briefly, MCF7 cells were transfected in 24 well plates with tracrRNA, crRNA and Cas9 plasmid. 72 hours after transfection, cells were plated in 15 cm dishes and continuously cultured until colonies formed. Colonies were recovered and profiled using PCR and Sanger sequencing to determine the presence of *CDH1* gene mutations. Loss of E-cadherin expression in the selected clone was confirmed using western blotting (Figure S5). The cell line and crRNA sequence are available upon request.

#### Total Lysate Preparation for Mass Spectrometry

Cells were plated in 100 mm dishes. Once confluent, media was discarded and cells were washed in PBS. Cells were lysed in a lysis buffer containing 2% SDS (Fisher Scientific), 20 mM Tris-HCl pH 7.5, 150 mM NaCl, 1 mM MgCl_2_, (Sigma-Aldrich) supplemented with protease inhibitor tablets (Roche) and phosphatase inhibitors (2 mM sodium orthovanadate, 10 mM sodium fluoride and 10 mM β-glycerophosphate) (Sigma-Aldrich). Lysates were subjected to sonication (Syclon ultrasonic cell disrupter), boiling (95°C, 5 min) and placed on ice for 10-15 min prior to centrifugation (14000 rcf, 10 min). The supernatant was transferred to fresh eppendorfs and samples were subsequently placed on ice for a further 10-15 min to allow the SDS to precipitate and re-centrifuged. Supernatant was transferred to fresh eppendorfs and protein concentration was measured using the Pierce BCA protein assay kit as per manufacturers instruction (Thermo Scientific), using a SpectraMax M3 (Molecular Devices). Once quantified, DL-DTT (DTT) was added to the lysates at a final concentration of 0.1 M DTT. Subsequently, lysates were boiled (95°C, 5 min). Detergent was removed from the lysates prior to MS analysis using the Filter Aided Sample Preparation (FASP) procedure incorporating Vivacon spin ultracentrifugation units with a molecular weight cutoff of 30 kDa (Sartorius)(Wisniewski et al., 2009). Briefly, 200 μl of urea buffer (Fisher Scientific) UA buffer (8 M urea in 0.1 M Tris-HCl pH 8.9) was added to 100 μg of cell lysate. Samples were added to the filter unit and centrifuged at 14000 rcf for 15 min. An additional 200 μl of UA buffer was added to the filter unit and re-centrifuged. Iodoacetamide (100 μl, 0.05 M prepared in UA buffer) was added to the filter units, incubated for 1 min on a thermomixer at 600 rpm and subsequently incubated in darkness for 20 min. Following the incubation period, filter units were centrifuged and washed twice with 100 μl of UA buffer followed by 2 washes with 100 μl of ABC solution (0.05 M NH_4_HC0_3_). After the final wash step, filter units were transferred to a new collection tube and a multi-step digestion method was employed as described by Wisniewski and Mann (Wisniewski and Mann, 2012). In the first instance, proteins were digested in a wet chamber overnight at 37°C using a solution containing Lys-C (Lysl Endopeptidase, Wako) and ABC buffer (1:50, enzyme to protein ratio). The following day, liberated peptides were collected by centrifugation and subsequent wash cycles with ABC buffer. Meanwhile, remaining proteins on the filter unit were digested using a solution containing Sequencing Grade Modified Trypsin (Promega) and ABC buffer in a wet chamber at 37°C for a minimum of 4 hr. Once again liberated peptides were collected by centrifugation and subsequent wash cycles with ABC buffer. The concentration of the Lys-C digests and Trypsin digests were measured using a NanoDrop 2000. In total, 10 μg of each digest was loaded onto activated handmade C18 StageTips as described previously (Rappsilber et al., 2003). StageTips were desalted with two 1% TFA wash cycles and bound peptides were eluted with 2 X 25 μl of 50% ACN/0.1% TFA. Final eluates were concentrated in the speed-vacuum centrifuge (Centri-Vap concentrator, Labconco to a final volume of ∼5 μl. Samples were then resuspended by adding 0.1% acetic acid, to a final volume of 15 μl and analyzed by mass spectrometry.

#### Mass Spectrometry

Mass spectrometry analysis was performed on a Q-Exactive mass spectrometer (Thermo Scientific), connected to a Dionex Ultimate 3000 (RSLCnano) chromatography system (Thermo Scientific) incorporating an autosampler. Five microliters of Lys-C/tryptic peptides was loaded onto a fused silica emitter (75μm ID, pulled using a laser puller (Sutter Instruments P2000)), packed with 1.8μ 120Å UChrom C18 packing material (NanoLCMS Solutions) and separated using an increasing acetonitrile gradient of 2 – 35%, with a 180 min reverse phase gradient at a flow rate of 250 nl/min. The instrument was operating in positive ion mode and with a capillary temperature of 320°C, coupled to a potential of 2300V applied to the column. Scan parameters for MS1 were as follows: Resolution 70,000, AGC 3e^6,^ MIT 60ms while scan parameters for MS2 were: Resolution 17,500, AGC 5e^4^, MIT 250ms, NCE 27.0, Isolation window 1.6m/z. The exclusion list parameters contained no entries and charge exclusion was set to un-assigned and singly charged. Both MS1 and MS2 were recorded as profile data. Data were acquired in automatic data-dependent switching mode, with a high-resolution MS scan (300-1600 m/z) selecting the 12 most intense ions prior to tandem MS (MS/MS) analysis. Each biological sample (n=3) was run in technical duplicate.

### Quantification and Statistical Analysis

#### Protein ID Matching

Identifiers in all protein-protein interaction networks, protein expression datasets, and validation sets were converted to ENTREZ gene IDs. In cases where a particular gene or protein could not be matched to an ENTREZ gene ID it was discarded from further analyses.

#### Protein Expression Data Processing

For the primary analysis we used the breast tumor proteomics dataset from the TCGA CPTAC project (Mertins et al., 2016). Only samples that passed the authors’ quality control (77 samples, 3 replicates, 3 controls) were used in our analysis. For validation we used two additional datasets – Tyanova et al (Tyanova et al., 2016) containing 40 tumor proteomes from diverse breast cancer subtypes, and Pozniak et al (Pozniak et al., 2016) containing 66 proteomes from primary luminal-type breast tumors or metastases. The dataset of Tyanova et al contains SILAC ratios which we converted using a log2 transformation prior to calculating correlations. For all proteomics datasets proteins absent in more than 40% of samples were discarded. As the average Pearson’s correlation between protein isoforms of the same gene was extremely high (0.95 in Mertins et al) multiple proteins mapping to the same gene were averaged into a single gene-level score. The resulting datasets contained profiles for 9,833 proteins (Mertins et al., 2016), 5,248 proteins (Tyanova et al., 2016) and 4,361 proteins (Pozniak et al., 2016).

#### Protein Interaction Network Assembly

We assembled an integrated protein interaction network from multiple sources. From the HINT database (Das and Yu, 2012) we included all co-complex interactions that were reported in at least two publications. From the BioGRID database (Chatr-Aryamontri et al., 2016) we included all protein-protein interactions in the multi-validated interactome – a network of interactions that were either observed in two experimental systems or in two separate publications. We augmented this set of high-confidence interactions with the result of four recent large-scale protein interactome mapping efforts (Havugimana et al., 2012, Hein et al., 2015, Huttlin et al., 2015, Wan et al., 2015). The resulting integrated network contained 83,656 interactions between 11,930 proteins.

#### Protein Complex Identification

Our goal was to identify sets of proteins (complexes) such that each complex consisted of a set of proteins whose expression profiles were highly similar across tumor profiles and that were densely connected on the protein interaction network. Other formulations are possible, but we chose to focus on disjoint complexes, such that each protein could only belong to a single complex. We did not require that every protein be assigned to a complex.

There are three components to our approach 1) choosing a score to evaluate the similarity of the expression profiles of a set of proteins 2) the identification of a similar score to evaluate the connectivity of a set of proteins on an interaction network, and 3) the identification of sets of proteins that score well on both datasets.

1) Scoring complexes using expression profiles

We calculate the Pearson’s correlation coefficient between each pair of expression profiles (A, B) and use this to compute a log-likelihood ratio that A and B belong to the same protein complex versus the likelihood that they are unrelated. This can formalized as follows:

LLR_expression_(A, B) = P_within_(A, B) / P_background_(A, B)

P_within_ is calculated using logistic regression trained on CORUM co-complexed pairs (Ruepp et al., 2010) as true positive examples. To prevent bias resulting from the large number of co-complex pairs falling within extraordinarily large complexes (e.g. Spliceosome, Proteasome, Ribosome) we exclude CORUM complexes containing more than 30 proteins from our training set. We assume a ratio of 300 negatives for every true positive, consistent with estimates of the size of the human interactome (Stumpf et al., 2008). Negative training examples are chosen randomly from the set of proteins with measured protein expression. P_background_ is the probability of observing the measured correlation between A and B in the set of all pairwise correlations.

For each set of proteins (S) we calculate the total LLR_expression_(S) as the sum of all LLR_expression_(A,B) scores for all unordered pairs (A,B) in the set S.

2) Scoring complexes using the protein-protein interaction network

For the protein-protein interaction network we sought to score each pair of proteins based on how likely they are to form part of the same complex. While direct protein-protein interaction provides an indication that two proteins may be part of a protein complex, previous work has demonstrated that taking into account the fraction of interaction partners shared by two proteins can provide additional support of co-complex membership (Bader et al., 2004, Goldberg and Roth, 2003). Based on this principle we assigned a weighted score to every pair of interacting proteins in our integrated network accounting for the proportion of interaction partners they share. This score was equal to a –log10 transformed p-value calculated from a hypergeometric test that assessed the significance of the number of interaction partners they shared. An advantage of this approach is that two proteins that interact with each other directly and share all of their interaction partners will be given a higher score than two proteins that interact with each other but have no other interaction partners in common.

As with the protein expression correlation, this score was transformed into log-likelihood ratio (LLR_interaction_) by comparing the probability of observing a particular score within a protein complex to the probability of observing it among all pairs of proteins. For each set of proteins (S) we calculate the total LLR_interaction_(S) as the sum of all LLR_interaction_(A,B) scores for all unordered pairs (A,B) in the set S.

3) Identifying complexes supported by both data sources

For each set of proteins we can assign a score LLR_integrated_(S), which is equal to the sum of LLR_expression_(S) and LLR_interaction_(S). We found that this LLR_interaction_ score predicted co-complex membership better than co-expression alone (Figure S1E).

Our challenge is the identification of sets of proteins with high LLR_integrated_ scores. As we are only interested in sets of proteins that score well on both resources we can restrict our search to those sets that have a positive LLR_interaction_ and a positive LLR_expression_ (i.e. we are only interested in sets of proteins that have highly correlated protein expression and are densely connected on the protein interaction network, not one or the other).

We identify high-scoring sets of proteins using an approach resembling agglomerative hierarchical clustering. Similar approaches have been used previously to identify complexes supported by genetic interaction and protein interaction networks in budding yeast (Bandyopadhyay et al., 2008) and also to identify complexes supported by the genetic interaction networks of two distinct yeast species (Ryan et al., 2012).

To initialize our clusters we first evaluate LLR_integrated_ for all pairs of proteins that directly interact in the protein-protein interaction network. We also evaluate scores for all possible 3-cliques (sets of three proteins that all interact with each other) in the protein-protein interaction network. The highest scoring pair or 3-clique is taken as an initial cluster, and all overlapping pairs or 3-cliques are then removed from consideration. The second highest scoring pair or 3-clique is then assigned as a cluster, and any overlapping pairs or 3-cliques removed from consideration. This continues until no pairs or 3-cliques with positive LLR_integrated_ scores remain. At the end of the process proteins that have not been assigned to any cluster are assigned to their own single element cluster. We then apply an iterative approach to improve these clusters. At each iteration we consider three possible moves – merging, removal and switching. Each pair of clusters (m_1_,m_2_) is evaluated for merging into a single cluster (m_1_ U m_2_) and assigned a score LLR_integrated_(m_1_,m_2_). For every protein in every cluster with multiple proteins we also calculate a LLR_remove_ score that reflects the change in the log likelihood resulting from removing that protein from the cluster, and an LLR_switch_ score that calculates the change in likelihood from switching a protein from one cluster to another. At each iteration max(LLR_merge_, LLR_remove_, LLR_switch_) is taken as the next move. To prevent the identification of clusters supported by only one data source (e.g. highly correlated expression but not densely connected on the protein interaction network) we only permitted moves in cases where the move resulted in an increase in the LLR score for both the expression and the protein interaction networks. Iterations continue until no move that increases the LLR score on both sources is identified. The end result is a list of clusters with an associated LLR score.

##### Estimating a Protein Complex False Discovery Rate

We assume that by chance some proteins that interact on the protein interaction network would have high co-expression scores and consequently we could identify clusters with positive LLR_expression_ and LLR_interaction_ scores. To remove potentially spuriously detected clusters we compared the clusters we identified to those identified using 100 randomized versions of the input - the same protein interaction network and expression set, but with the gene IDs on the expression set shuffled. These randomized networks allowed us to empirically estimate the False Discovery Rate as we could see for a given LLR_integrated_ score how many genes would be assigned to complexes in the randomized networks compared to the genes assigned to complexes in the real network. We chose an FDR of 10% for defining the BrCa-Core set of complexes.

#### Protein Complex Evaluation

To assess the overlap between BrCa-Core complexes and existing annotation sets (CORUM complexes, Gene Ontology Cellular Compartment, Gene Ontology Biological Process) we used the gProfiler tool (Reimand et al., 2016). Only genes present in both the protein-interaction network and the tumor proteome expression were used as the background list or this enrichment. Multiple testing correction was performed using the default g:SCS approach (Reimand et al., 2016).

We calculated the average Pearson correlation between complex subunits using the dataset of Tyanova et al (Tyanova et al., 2016) and Pozniak et al (Pozniak et al., 2016). For this analysis we excluded pairs of proteins whose genes reside on the same chromosome to avoid high correlation resulting solely from co-amplification/co-deletion events. For the shRNA data from (Marcotte et al., 2016) we calculated the Pearson’s correlation of co-complexed pairs using the zGARP profiles of 77 breast cancer cell lines.

From the STRING database (Szklarczyk et al., 2017) we extracted pairs of proteins that are frequently mentioned together in the literature (textmining score > 250) and that tend to co-occur in a significant pattern across species (cooccurence score > 0). Fisher’s exact test was used to assess the significance of the overlap between the BrCa-Core co-complexed pairs and these reference datasets.

#### Identifying Subtype Specific Complex Expression

To identify protein complexes differentially expressed in specific breast cancer subtypes we used a variant of the 1D annotation enrichment test proposed by Cox and Mann (Cox and Mann, 2012). For each protein we calculate the difference between the median expression of samples from a specific subtype and the median expression of samples from all other subtypes combined. We then applied a Mann Whitney test to these median differences to see if the members of a given protein complex are among the most significantly differentially expressed proteins in a particular subtype (i.e. to see if all/most complex members are at one end of a ranked list of differentially expressed proteins). This test is performed in a two-sided fashion to identify complexes that are either over- or under-expressed in specific subtypes. All protein complexes with more than two members are tested for differential expression in all three subtypes. We correct for multiple-hypothesis testing using the Benjamini and Hochberg approach (Benjamini and Hochberg, 1995), and identified a set of 82 differentially expressed complexes at an FDR of 10%. We then tested these complexes for differential expression in the dataset of Tyanova et al at the same FDR. As not every BrCa-Core complex is represented by multiple members in Tyanova et al we could test only 59 of these associations. The s-score (Cox and Mann, 2012) was used to measure the effect size of the association between protein complex expression and subtype, and Spearman’s correlation was used to assess the concordance of effect sizes between the associations identified in the Mertins et al data and those in Tyanova et al.

#### Mutation, Copy Number, mRNA and RPPA Data

Sequence, copy number and mRNA expression profiles for were all obtained through the cBioPortal (Breast Invasive Carcinoma, TCGA Provisional) (Gao et al., 2013). To identify associations between mutation/deletion and protein abundance we annotated all tumor samples according to whether or not they featured mutations or deletions in each of the genes coding for proteins in the BrCa-Core set. For copy number profiles we considered genes to be homozygously deleted in a specific sample if they had a GISTIC score of -2 and hemizygously deleted if they had a GISTIC score of -1. We considered genes to be mutated if they harbored a non-synonymous missense mutation, splice-site mutation, an insertion or deletion, or a nonsense mutation. For the RPPA analysis and mRNA expression analysis presented in Figure 4 we used the Z score normalized expression levels available through the cBioPortal (Gao et al., 2013).

#### Associating Mutation/Deletion with Complex Expression

To assess how genetic variants altered the overall abundance of protein complexes to which they were associated, we first converted the quantitative measurements of protein expression into rank orders. In each sample we then calculated the mean rank of all complex members, and tested if samples harboring the alteration of interest displayed lower mean rank than samples without the alteration using a one-sided Mann Whitney U test. The mean rank was calculated with the deleted gene excluded (e.g. COPS3 was excluded when calculating the mean rank of the COP9 signalsome). For deletions (homozygous or hemizygous) complex members on the same chromosome as the altered gene were also excluded from the mean rank calculation.

#### MCF7 Proteomic Analysis

Mass spectra were analyzed using MaxQuant software (version 1.5.0.25)(Cox and Mann, 2008) containing the in-built Andromeda search engine to identify the proteins from a human database (Uniprot HUMAN, release 2012_01) containing 20,242 entries. Default parameters were selected in MaxQuant with the exception of the selection of the relevant enzyme, (LysC and Trypsin digests were separated between parameter groups). For database searches, the precursor mass tolerance was set to 20 ppm for first searches and 4.5 ppm for main Andromeda search. The search included a fixed modification of Carbamidomethyl (C) and variable modifications of Oxidation (M);Acetyl (Protein N-term). Label free quantification with a minimum ratio count of 2 was selected, the maximum number of missed cleavages was set at 2 and minimum peptide length was set to 7 amino acids. An FDR of 0.01 was set for peptide and protein identifications. Match between runs was selected with a matching time window of 0.7 min and alignment time window of 20min. The presence of reverse and contaminant identifications were removed from the dataset.

##### Differential Expression Analysis

Proteomic profiles were generated for three biological replicates of the parental (*CDH1* wild-type) and *CDH1*-defective cell lines. Two technical replicates were obtained for each biological replicate and these were averaged prior to further analysis. Missing values were imputed using the minimum observed intensity for each sample, based on the assumption that missing proteins could be absent or below the detection threshold of the instrument. Log2 transformed LFQ (Label Free Quantification) values were used for analysis. A two-sided heteroscedastic t-test (Welch’s t-test) was used to identify differentially expressed proteins and the Benjamini-Hochberg approach was used to estimate the False Discovery Rate (Benjamini and Hochberg, 1995).

### Data and Software Availability

The MCF7 CDH+/- mass spectrometry proteomics data have been deposited to the ProteomeXchange Consortium via the PRIDE partner repository with the dataset identifier PXD007543. Python code for the complex identification method, along with necessary input data, can be obtained from: https://github.com/cancergenetics/brca-core

## Author Contributions

C.J.R. conceived and designed the study, wrote all code, and drafted the manuscript. D.M. and S.K. performed and analyzed proteomics experiments. I.B. and C.J.L. created the CDH1 model. All authors aided interpretation of the results, provided input on the manuscript, and read and approved the final manuscript.

## Supplemental Information

Document S1. Figures S1–S5

Table S1. BrCa-Core Complex Composition, Related to Figure 1

Table S2. Subtype-Specific BrCA-Core Complex Expression, Related to Figure 2

Table S3. Collateral Loss Events Associated with Hemizygous Deletion, Related to Figure 3

Table S4. MCF7 CDH1 Isogenic Protein Expression Profiles, Related to Figure 4

Document S2. Article plus Supplemental Information
