# Supplementary material for: A Compendium of Co-regulated Protein Complexes in Breast Cancer Reveals Collateral Loss Events
Source: Cell Syst. 2017 Oct 25;5(4):399–409.e5. doi: 10.1016/j.cels.2017.09.011 (PMC5660599; doi:10.1016/j.cels.2017.09.011)
Supplement: Document S1. Figures S1–S5 [file mmc1.pdf]

Cell Systems, Volume *5*

Supplemental Information

A Compendium of Co-regulated Protein Complexes in Breast Cancer Reveals Collateral Loss Events

Colm J. Ryan, Susan Kennedy, Ilirjana Bajrami, David Matallanas, and Christopher J. Lord

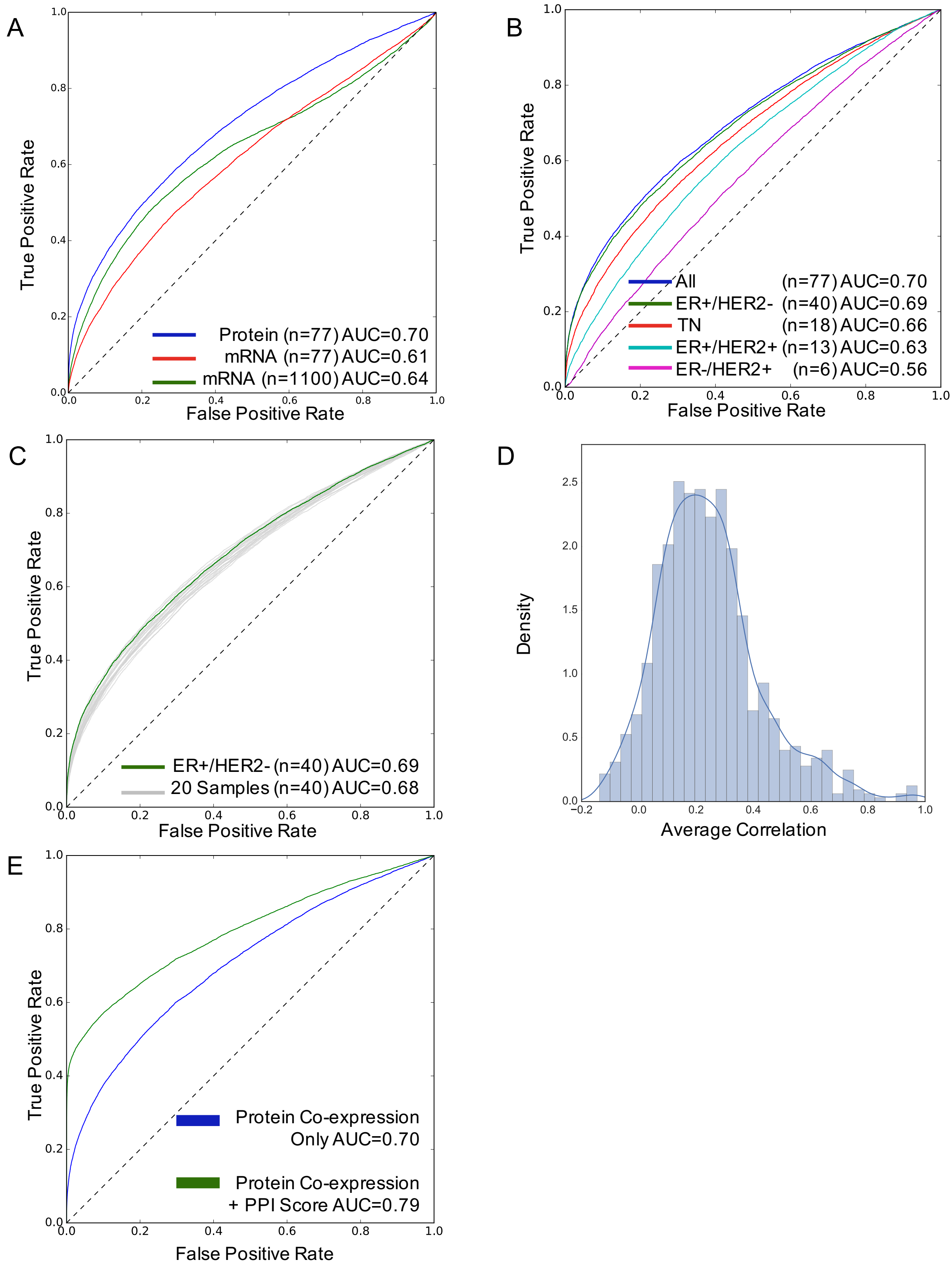

Figure S1. Protein co-expression is predictive of co-complex membership. Related to Figure 1. **A)** ROC curve showing the ability of co-expression to predict co-complex membership. Blue line shows ROC curve derived from correlation calculated using protein expression profiles from 77 tumors, while the red line indicates that derived from mRNA expression profiles for the same samples. The green line indicates

the co-expression correlation calculated using a larger set of 1,100 mRNA expression profiles. **B)** ROC curve comparing co-expression calculated over all subtypes combined with co-expression calculated over samples from individual subtypes **C)** ROC curve comparing co-expression calculated over all samples from a specific subtype (ER+/HER2-) to the same number of samples selected at random from all subtypes. **D)** ROC curve comparing co-expression integrated with protein-protein interaction score ($LLR_{integrated}$) to co-expression alone for predicting co-complex membership. **E)** Histogram showing the average protein expression correlation within CORUM complexes. Only the average correlations from complexes with at least three members are shown in the plot.

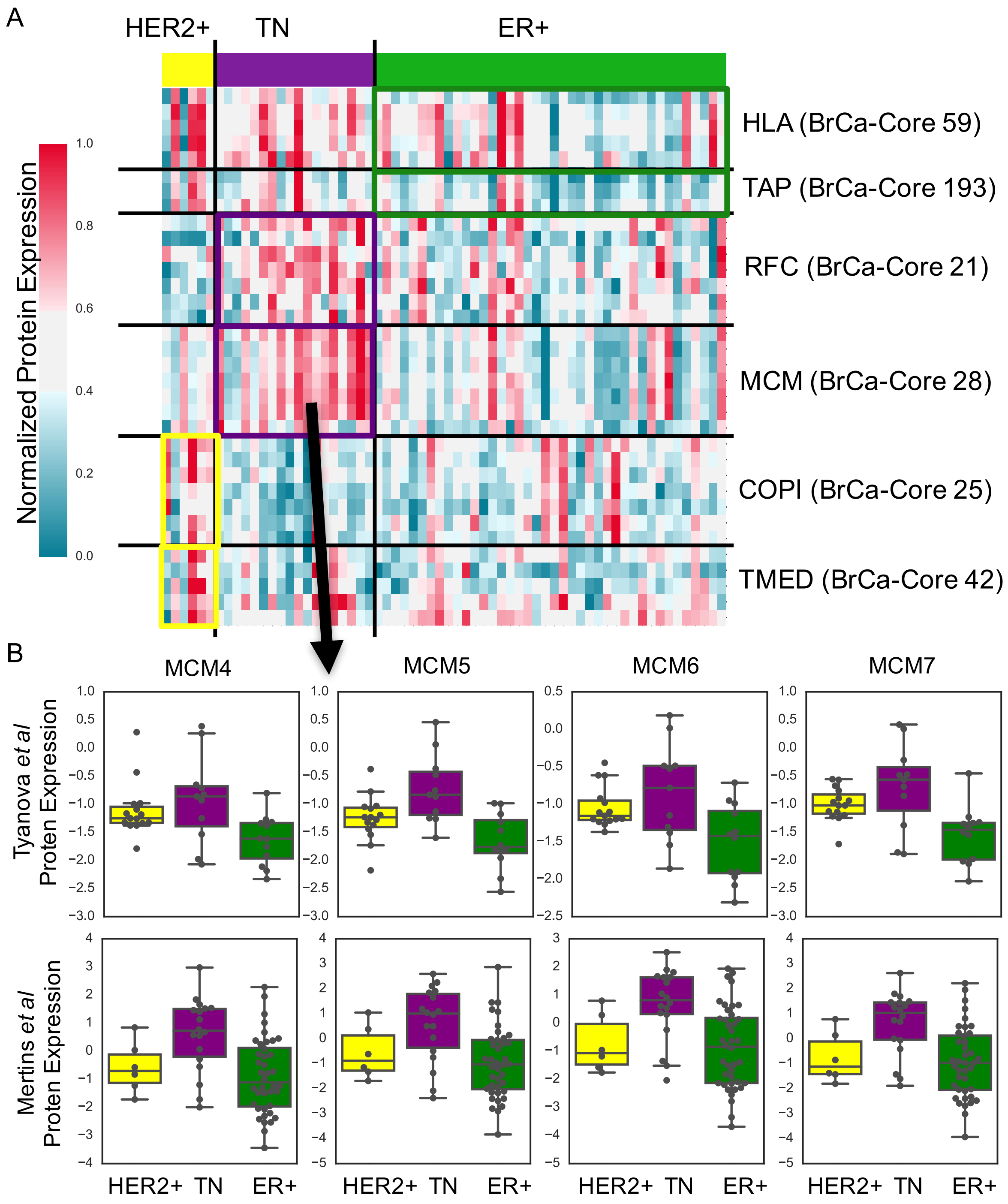

Figure S2. Subtype specific complex expression. Related to Figure 2. **A)** Heatmap displaying expression levels of specific protein complexes. Tumor samples are grouped according to subtype (using IHC), indicated on top of the heatmap. Genes are grouped into specific complexes indicated on the right of the heatmap. All expression levels have been normalized such that the maximum expression level is 1 and minimum is 0. Shown are the expression levels from Mertens *et al* (used for discovery). Expression levels for Tyanova *et al* (used for validation) are shown in Figure 2. **B)** Boxplots displaying the subtype specific protein expression levels of selected subunits of the MCM complex (BrCa-Core 28) in the Tyanova *et al* dataset

(top) and TCGA dataset (bottom). These box plots show median and interquartile range and are colored according to sample subtype.

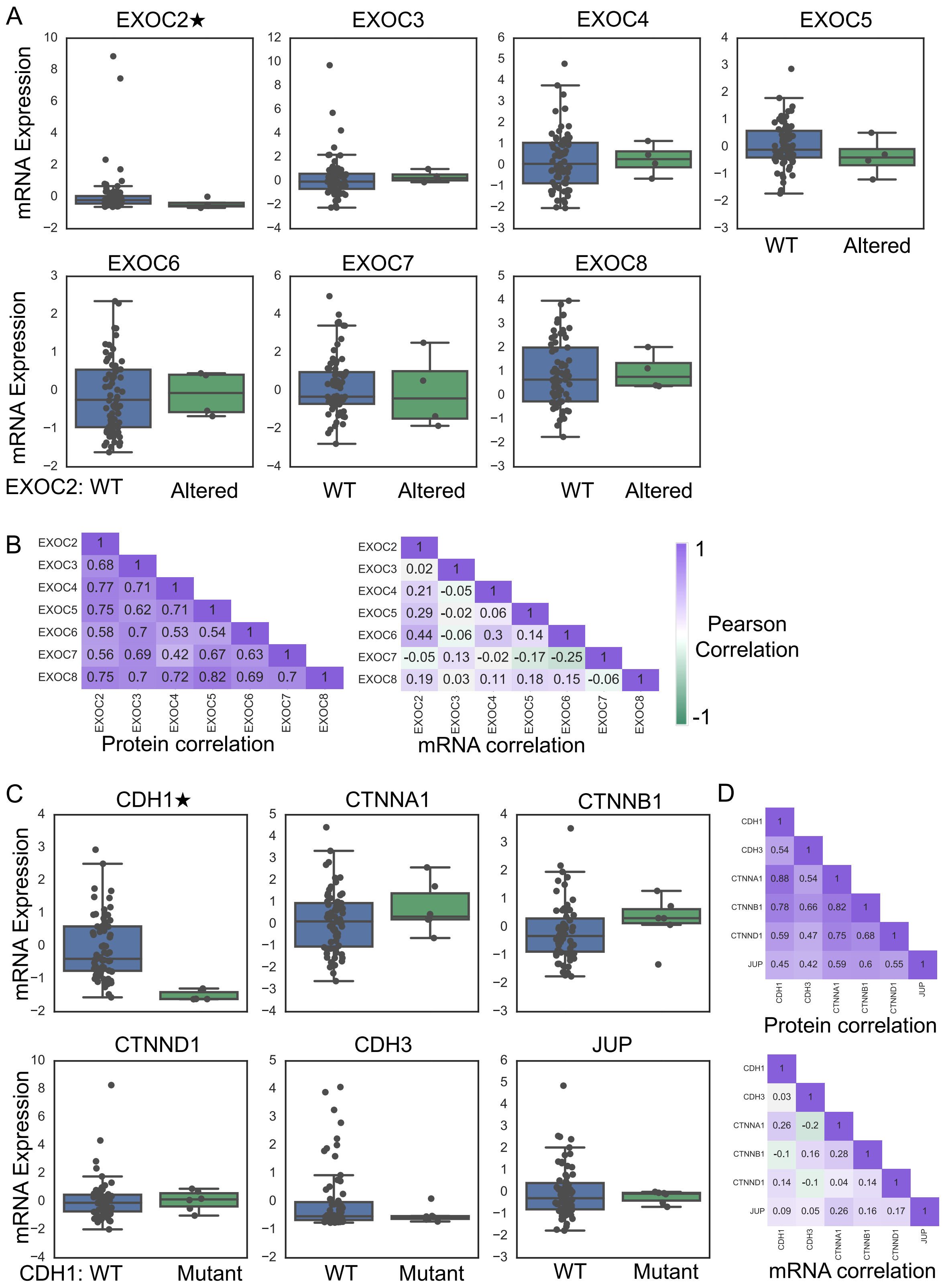

Figure S3. Subunit loss is not associated with a reduction in protein complex mRNA expression. Related to Figure 3. **A)** Mutation or deletion of *EXOC2* is not associated with a reduction in mRNA expression of the exocyst complex (BrCa-Core 27). Boxplots display the mRNA expression of different subunits partitioned according

to *EXOC2* status. Each box plot shows the median and interquartile range. Genes marked with a star indicate those whose mRNA abundance is significantly lower (one-sided Mann Whitney test, $p<0.05$) in samples with *EXOC2* mutation/deletion. **B)** Heatmap showing the Pearson's correlation between the subunits of the exocyst complex calculated using protein expression (left) and mRNA expression (right). **C)** Mutation of *CDH1* is not associated with a reduction in mRNA expression of the adherens junction complex (BrCa-Core 30). Legend as for A. **D)** Heatmap showing the Pearson's correlation between the subunits of the adherens junction complex calculated using protein expression (top) and mRNA expression (bottom)

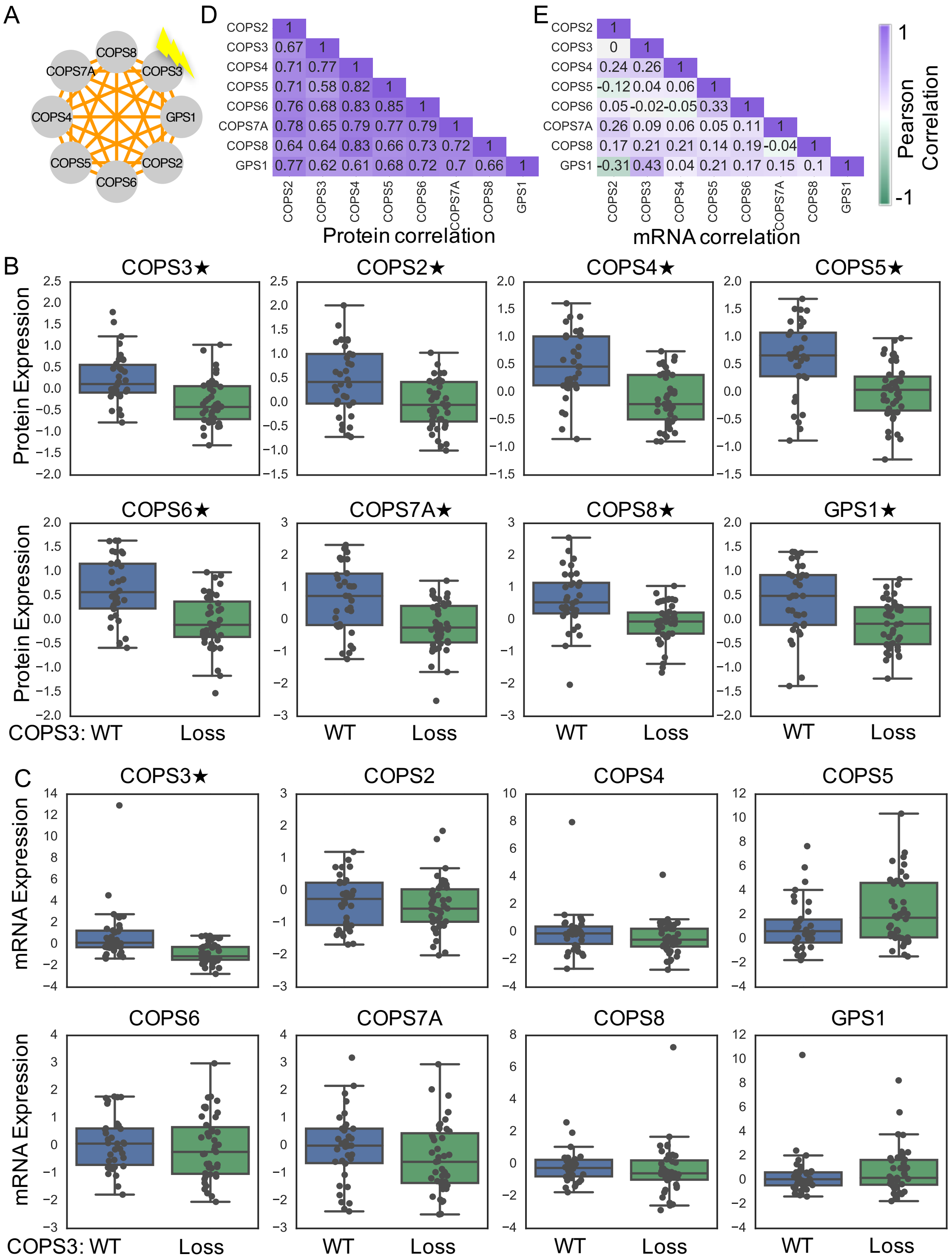

Figure S4. COPS3 hemizygous deletion is associated with reduced protein expression of the COP9 signalosome. Related to Figure 3. A) The COP9 signalosome identified in BrCa-Core (BrCa-Core 17) with COPS3 highlighted. B) Hemizygous deletion of *COPS3* is associated with a reduction in protein abundance of all subunits of BrCa-Core 17. Boxplots display the protein abundance of different subunits partitioned according to *COPS3* status. Each box plot shows the median and interquartile range. Genes marked with a star indicate those whose proteomic

abundance is significantly lower (one-sided Mann Whitney test, $p<0.05$) in samples with *COPS3* hemizygous deletion. C) Hemizygous deletion of *COPS3* is not associated with a reduction in mRNA expression of most subunits of BrCa-Core 17. Boxplots display the mRNA expression of different subunits partitioned according to *COPS3* status. D) Heatmap showing the Pearson's correlation between the subunits of the COP9 signalosome calculated using protein expression. E) Heatmap showing the Pearson's correlation between the subunits of the COP9 signalosome calculated using mRNA expression

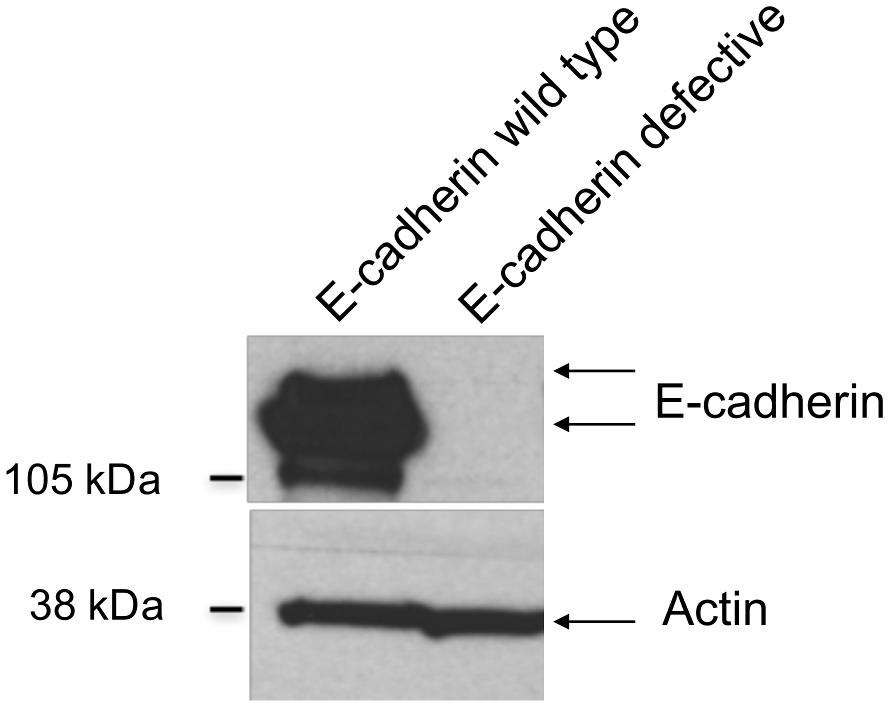

Figure S5. *CDH1* mutant MCF7 cells display loss of E-cadherin protein expression. Related to Figure 3. Western blot showing E-cadherin expression in parental MCF7 cells ("E-cadherin wild type") and the *CDH1* mutant clone ("E-cadherin defective") used for mass spectrometry analysis. Upper E-cadherin band represents a glycosylated isoform, the lower band representing the non-glycosylated form. For this analysis, 50 ug of whole cell lysates were electrophoresed on Novex 4–12% gradient bistris pre-cast gels (Invitrogen) and immunoblotted overnight at 4°C with antibodies listed in the Key Resources.
